# Expression of Hyaluronan in human tumor progression

**DOI:** 10.1186/1477-3163-5-2

**Published:** 2006-01-10

**Authors:** Rajeev K Boregowda, Hitesh N Appaiah, Manjunath Siddaiah, Sunil B Kumarswamy, Sunila Sunila, Thimmaiah KN, KarunaKumar Mortha, Bryan Toole, Shib d Banerjee

**Affiliations:** 1 Department of Pathology, Washington University School of Medicine, St. Louis, MO 63110, USA; 2 Department of Biochemistry, University of Mysore, Mysore, India; 3 Department of Chemistry, University of Mysore, Mysore, India; 4 Department of Pathology, J.S.S Medical College, Mysore, India; 5 Department of Cell Biology and Anatomy, Medical University of South Carolina, Charleston, SC 29425, USA; 6 Molecular Pharmacology, St Jude Children's Research Hospital, Memphis, TN 38105 USA

## Abstract

**Background:**

The development and progression of human tumors is accompanied by various cellular, biochemical and genetic alterations. These events include tumor cells interaction with extracellular matrix molecules including hyaluronan (HA). Hyaluronan is a large polysaccharide associated with pericellular matrix of proliferating, migrating cells. Its implication in malignant transformation, tumor progression and with the degree of differentiation in various invasive tumors has well accepted. It has been well known the role HA receptors in tumor growth and metastasis in various cancer tissues. Previously we have observed the unified over expression of Hyaluronic Acid Binding Protein (HABP), H11B2C2 antigen by the tumor cells in various types progressing tumor tissues with different grades. However, the poor understanding of relation between HA and HA-binding protein expression on tumor cells during tumor progression as well as the asymmetric observations of the role of HA expression in tumor progression prompted us to examine the degree of HA expression on tumor cells vs. stroma in various types of human tumors with different grades.

**Methods:**

In the present study clinically diagnosed tumor tissue samples of different grades were used to screen the histopathological expression of hyaluronan by using b-PG (biotinylated proteoglycan) as a probe and we compared the relative HA expression on tumor cells vs. stroma in well differentiated and poorly differentiated tumors. Specificity of the reaction was confirmed either by pre-digesting the tissue sections with hyaluronidase enzyme or by staining the sections with pre-absorbed complex of the probe and HA-oligomers.

**Results:**

We show here the down regulation of HA expression in tumor cells is associated with progression of tumor from well differentiated through poorly differentiated stage, despite the constant HA expression in the tumor associated stroma.

**Conclusion:**

The present finding enlighten the relative roles of HA expression on tumor vs. stroma during the progression of tumors.

## Background

Hyaluronan (HA) is a ubiquitous component of the extracellular, pericellular and intracellular matrices that mediates cell proliferation, migration, developmental, differentiation, morphogenetic processes in the developing organs, inflammation and tumor development [1]. Hyaluronan is structurally a simple primitive molecule. It is a large linear glycosaminoglycan composed of uniformly multiple repeats of β-1,3 N-acetylglucosaminyl and β-1,4 glucuronide disaccharide unit mainly produced by the stromal cells and it is distributed in various compartments of tissues [10]. HA's physicochemical properties contribute to the formation of space which aids in the tumor cells migration and infiltration of newly formed blood vessels [18]. The presence of HA is well evidenced in the cytoplasm and nuclei of the cells in a number of tissues *in vivo *to modulate the cell behavior by interacting with specific cell surface receptor- hyaluronic acid binding proteins (HABPs) known as Hyaladherins. This phenomenon also plays an important role in maintaining and stabilizing the structural integrity of extracellular matrices [2-4]. Recent studies have shown that there are a number of HA-binding proteins that may be important in the regulation of cell cycle or gene transcription [5-8]. It is an established fact that most malignant tumors such as colon carcinoma [11], breast carcinoma [12] gliomas [13], lung carcinomas [14,15] and wilms' tumors [16] contain elevated levels of HA than their normal counterparts. The distribution of HA in normal esophagus, stomach, colon and in adenocarinomas of these tissues was ruled out as a possible indicator for differentiation process [17]. The increased metastasis behavior of mouse mammary carcinoma [19] and melanoma cells [20] has been related to increase HA synthesis by tumor cells.

In tumor tissues, HA promotes tumor metastasis by opening up spaces for tumor cells to migrate and actively supports tumor cell migration by interacting with cell surface HA receptors [47]. Previously, our immuno histopathological detection of the continuous over-expression of HA-binding protein in correlation with tumor progression in human tumors of different origin with different grades identified the role of HA-binding protein as a prognostic indicator [9]. In this context, one might assume the down- regulation of tumor cell surface and intracellular HA with respect to tumor progression in relation with increased expression of Hyaluronidase enzyme by the tumor cells. This has raised the question about the fate of HA on the tumor cells and its role in tumor progression and invasion in to the stroma. In accordance with this concept it has been reported the high expression of HA in tumor associated stroma and increased HYAL-1 (Hyaluronidase) enzyme synthesis on tumor epithelial cells in high-grade prostate carcinoma [48]. The weak correlation between tumor cell hyaluronan and its perineural invasion as well as the prognostic value of stromal hyaluronan has been reported with metastasis in prostate cancer [49]. Despite the reports concerning the role of tumor vs. stromal hyaluronan in tumor progression, there was no clear evidence of its role in all the possible human tumors. In the present study we have screened the twenty one different types of progressing tumors of epithelial carcinomas, sarcomas and carcinomas of different origins with a specific probe for the localization and distribution of hyaluronan in tumor epithelia and intra-tumoral regions as well as benign areas. The results showed considerable up-regulation of hyaluronan in well differentiated tumors and associated stroma irrespective of their origin, while it is down-regulated in poorly differentiated tumors by retaining constant stromal hyaluronan expression. This study served as the explanation of the role of HA expression in tumor progression and invasion process as well.

## Methods

The study group consisted of 256 different samples. The samples were collected after surgery from cancer patients of different age groups at a local hospital between 1995 and 2002 after the approval of ethical committee from the hospital. The case history and diagnosis undertaken were recorded during sample collection. All the samples showed low to high-grade tumors and both malignant and normal areas were examined in all the cases. The tissues from biopsies were fixed as soon as possible in 10% buffered formalin and processed by the standard procedure. Paraffin blocks were made and 3 to 5 micron sections were cut and used for the histochemical analysis of HA. Specimens were selected based on the presence of both benign and malignant histology, present within the same tumor sections. All tissue sections were initially stained with hematoxylene and eosine method to evaluate the grades of the tumors. In all cases, routine independent pathological diagnosis was made in the pathology laboratory. Malignant tumors were classified as well differentiated (grade-I); moderately differentiated (grade-II); and poorly differentiated (grade-III) based on their level of differentiation.

### Localization of hyaluronan

Histochemical localization of hyaluronan was carried out by using a biotinylated HA-affinity probe. The tissues were incubated with the biotinylated probe which consisted of a mixture of cartilage proteoglycan core protein and link protein (kindly provided by Charles Underhill; Department of Anatomy and Cell Biology, Georgetown University and by Dr Bryan Toole; Department of Cell Biology and Anatomy, Medical University of South Carolina, Charleston, USA and purchased from Calbiochem, U.S.A.) at a concentration of 5 μg/ml which recognizes hyaluronan in tissues. Hyaluronan staining from 5 days old chicken embryonic limb tissue section (that has been processed like all other human tissues) was used as a positive control. The specificity of HA staining was confirmed by incubating tissue sections with either streptomyces or bovine testicular hyaluronidase (50 U/ml in acetate buffer pH 5.0, overnight at 37°C) prior to the incubation with the HA-affinity probe. In the second set of experiments the specificity of HA staining was additionally confirmed by staining the tissue sections with a pre-incubated complex of biotinylated PG with 100 μg/ml hyaluronan or 300 ug HA-oligomers. In all the cases the probe was applied in PBS containing 1% BSA for 2 hours at room temperature, the slides were washed in PBS and processed as described below.

### Chromogen treatment

In all cases, the slides were subsequently incubated with streptavidin-horseradish peroxidase conjugate (Bangalore Genei, Karnataka, India) for 1 hour at room temperature. The color reaction was developed for 15 minutes using diaminobenzidine hydrochloride (DAB) in 0.1 M Tris-Hcl pH 7.4. The substrate solution was freshly prepared by dissolving 6.0 mg DAB in 15 ml of 0.1 M Tris buffer, then 4.5 μl of 30% H_2_O_2 _was added just before use. The slide was rinsed in distilled water, sequentially dehydrated in graded alcohol and mounted in DPX mounting media. Photographs were taken with a Leica Photovat microscope.

## Results

### Hyaluronan localization in embryonic limb bud

**Figure 1:**
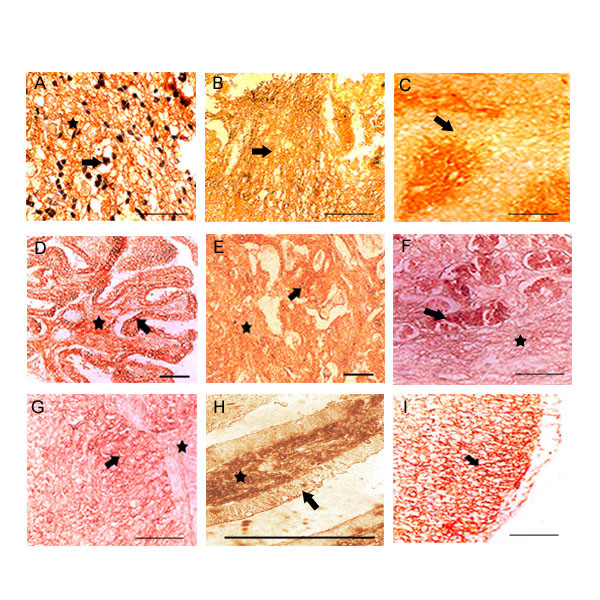
**HA expression in well differentiated tumors: **Only 8 examples are shown. **Top row, (A, B, C) **represents cerebral astrocytomas, ganglioglioma and salivary gland tumor. In astrocytomas neuroglial fibers and the astrocyte membrane are highly reactive for HA. Neoplastic nerve cells with extensive fibrillar processes are HA positive in ganglioglioma. Benign epithelia surrounding the tumor cells in salivary of showed low HA reaction. **Middle row, (D, E, F) **represents papillary carcinoma of thyroid, infiltrating breast and stomach tumor. Thyroid tumor showed intense HA staining at intraepithelial and in surrounding stromal components. High level HA expression was observed in tumor cells and surrounding stroma in case of infiltrating breast tumor. Tumor epithelia of stomach showed strong HA reaction. **Bottom row, (G, H, I) **represents Transitional epithelia of urinary bladder, descending colon and 5 days old chicken embryo limb. Transitional epithelia of urinary bladder showed intense staining at intra-epithelial and in surrounding tumor components. Invasive colon tumor cells showed strong HA reaction. 5 days chicken embryo limb was used as positive control, where the mesoderm is intensely positive for HA. Arrow shows the tumor cells and the asterisk shows stroma. Scale bars, 50 μm.

In the present study we have used 5 days chicken limb bud as an illustrative model and as a positive control to demonstrate the presence of hyaluronan. It is well known that early limb mesoderm cells are surrounded and separated by a hyaluronan rich matrix^33^. Fig 1 (I) shows the presence of highly enriched hyaluronan positive mesodermal cells in the chick embryo limb bud.

### Hyaluronan expression in well differentiated tumors

We have screened more than 20 different well differentiated tumors. The tumor tissue sections were deparaffinized, processed and stained with b.PG probe as explained in the materials and method section. There are 8 examples shown in Fig. 1. Astrocytoma, salivary gland, thyroid, infiltrating breast, stomach, urinary bladder and colon tumors are well differentiated (grade 1) (Fig 1A,C,D,E,F,G,H) and ganglioglioma (grade II) (Fig. 1B) In all the tumors intense hyaluronan staining was found both in the tumor cells, intra-tumoral and in the associated surrounding stroma.

### Hyaluronan expression in poorly differentiated tumors

**Figure 2:**
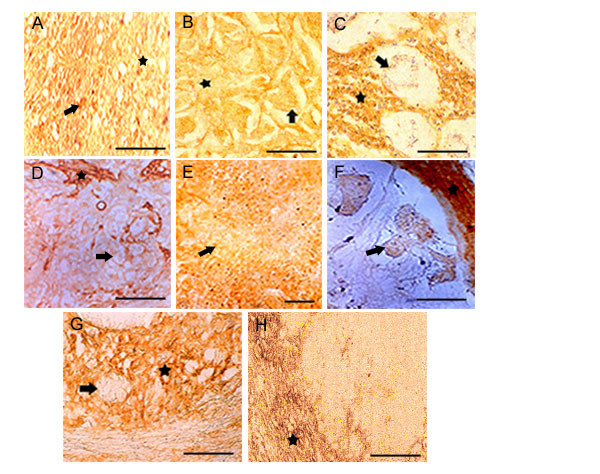
**HA expression in poorly differentiated tumors: **Only 8 examples are shown. **Top row, (A, B, C) **shows disappearance of HA stain from the tumor epithelia. Fibrillar astrocytomas show intense reaction while astrocytes are negative. Infiltrating breast and stomach tumor epithelia shows the loss of HA while the stromal components shows intense HA stain. **Middle row, (D, E F) **shows the loss HA expression in urinary bladder, pancreas (tumor epithelia) and caecum tumor epithelia while the stromal areas from urinary bladder and caecum were enriched with HA positive materials. **Bottom row, (G, H) **represents prostate and ovary. Prostate is showing numerous invading tumor cells in stromal areas. Only the stromal components are stained with b.PG. Ovary shows total absence of HA expression in tumor cells while the stroma is enriched with HA. Arrow shows the tumor cells and the asterisk shows stroma. Scale bars, 50 μm.

Out of 20 tumors studied we have presented 8 examples of highly advanced poorly differentiated tumor tissues here. The tissues were astrocytomas, infiltrating breast, stomach, gall bladder, pancreas, caecum, prostate, ovary (Fig. 2A to 2H). In all cases of epithelial carcinomas or sarcomas, the tumor cells showed almost complete lack of hyaluronan stain. However, the intratumoral and stromal areas showed moderate hyaluronan stain.

### Specificity of b.PG for hyaluronan staining

**Figure 3:**
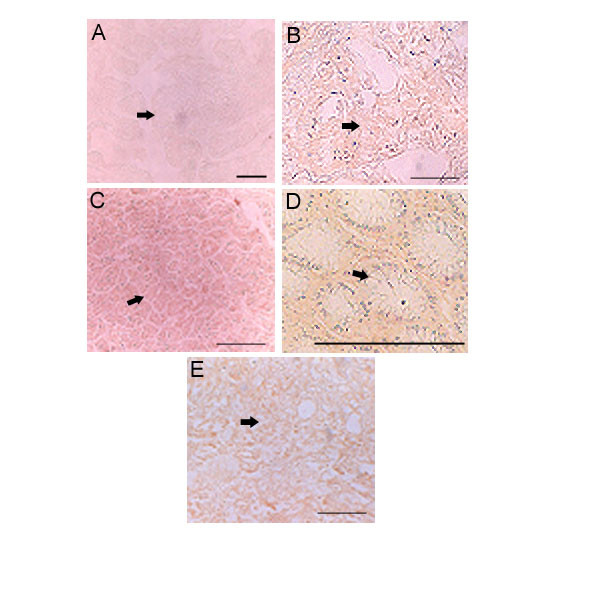
**Specificities of b.PG for HA staining: **Only 5 examples are shown. **A. **Thyroid, **B. **Infiltrating breast, **C. **Pancreas, **D. **Colon and **E. **Urinary bladder. All tumors are negative for Hyaluronic acid under different control experimental condition (explained in the material and methods). Scale bars, 50 μm.

The tumor tissues were either treated with hyaluronidase at 50 units/ml or bPG pre-absorbed with 300 μg/ml hyaluronan-oligomers. Irrespective of their origin the tumor showed negative staining of HA in the tumor epithelia or stromal area. Fig. 3A to 3E. shows Thyroid, Infiltrating breast, Pancreas, Descending Colon and Urinary bladder with the lack of HA staining.

### Hyaluronan expression in stroma

**Figure 4:**
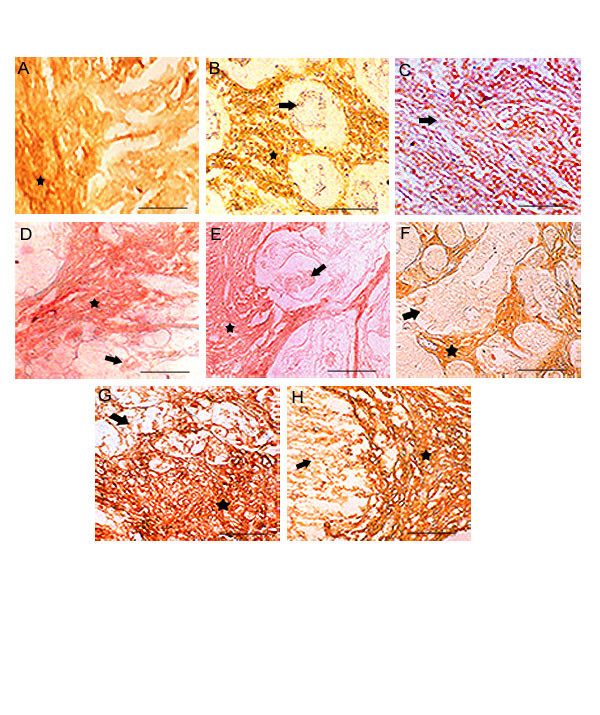
**HA staining in poorly differentiated stromal areas: **Top row, **(A, B, C) **represents breast, stomach and gallbladder. Middle row, **(D, E, F) **represents colon, caecum and prostate. Bottom row, **(G, H) **represents urinary bladder and renal cell carcinoma. The stromal and intratumoral areas are highly positive for HA in all tumors, where as the loss of HA expression observed in the tumor cells. Arrow shows the tumor cells and the asterisk shows stroma. Scale bars, 50 μm.

Intense hyaluronan staining was observed in intra and peritumoral areas of all the well differentiated tumors when compared to the benign areas. Carcinomas from astrocytomas, gangliogliomas, thyroid, breast and salivary showed intense accumulation of hyaluronan in intratumoral areas. Highly aggressive poorly differentiated tumors of different origins showed moderate to low levels of stromal hyaluronan in the immediate vicinity the tumor cells (Fig. 4A to 4H). High levels of stromal staining in highly -differentiated and moderate staining in poorly differentiated tumors stroma may indicate the role intra-tumoral and tumor associated stromal components in maintaining the progressive nature of the tumors.

### Tumor cell associated hyaluronan expression

**Figure 5:**
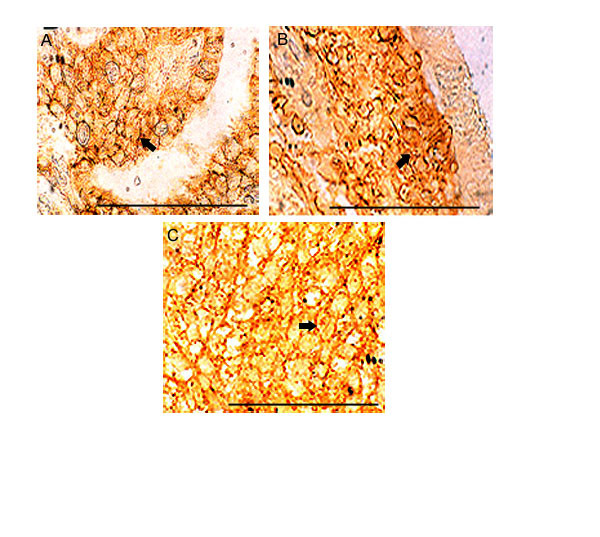
**HA on tumor epithelial cell surfaces: **Three examples are shown. **(A, B, C)**. **A. **Thyroid, **B. **Descending Colon, **C. **Urinary bladder. Tumors derived from well differentiated stage. Only 3 examples are shown. High accumulation of HA reactive materials observed on the cell surfaces of all the tumors. Arrows shows the cell surface. Scale bars, 50 μm.

Epithelial cells of benign areas were mostly hyaluronan negative. In the 20 tumors studied, epithelial cells surface were highly positive for HA in the well differentiated than in the poorly differentiated tumors. Only 3 examples are illustrated in Fig (5A,B and 5C) demonstrates the localization of hyaluronan on the plasma membrane.

## Discussion

The purpose of the present study was to investigate the HA expression in tumor cells and in intratumoral stromal areas in different types of tumor tissues with different origins and to reveal the possible role of HA involvement with progressive malignant behavior. Our results from 23 tumor tissues of different grades showed the down-regulation of HA expression in tumor epithelial cells during later stages of all types of malignancies irrespective of their origin. On the contrary, all highly differentiated tumors such as Astrocytomas, gangliogliomas, thyroid, lung, salivary, breast, esophagus, stomach, pancreas, colon, rectum, gall bladder, kidney, urinary bladder, prostate, testes, ovary, medulloblastoma, osteosarcoma, endocervix and non-Hodgkin's lymphoma expressed increased amount of HA in both tumor epithelia and the intratumoral areas.

Considerable evidence has been documented the significance of hyaluronan expression during human tumor progression where it was frequently observed in elevated levels around tumor cells facilitating tumor migration and proliferation [20]. High levels of HA expression correlates with poor tumor differentiation and poor survival rates [11,12]. Tumor cells differentiation during malignant transformation in bronchial and squamous cell carcinoma demonstrated the changes in HA distribution [15]. Albeit many tumor cells are enriched in HA [26], they nonetheless showed considerable variations in HA expression depending on their origin as well as on the histological type of the tumors [17]. Elevated levels of HA are found in most carcinomas of squamous-cell origin, colorectal epithelial [11], ovarian [21] and breast [22]. On the other hand loss of HA in progressive carcinomas from laryngeal, esophageal, colorectal and cutaneous melanomas [27] was observed. This generalization, and many years of investigations with conflicting reports on the spatial distribution of hyaluronan in early and late malignancy have increased the depth of the dilemma about the role of HA during tumor progression.

This dilemma along with our previous observation of linear over expression of HA-binding protein on the tumor cells during tumor progression prompted us to investigate more clearly the spatial and temporal expression of HA on both tumor cell surfaces and stroma during the progression of tumor to understand its possible role during tumor progression. We have concentrated specifically on well and poorly differentiated tumors from carcinomas, sarcomas and various squamous cell carcinomas. Our results from the present investigation clearly showed a total absence of hyaluronan on tumor epithelia in the poorly differentiated tumor as shown in figure 2, except in astrocytoma (fig. 2A) and a definite trend in the continued expression of hyaluronan in the intratumoral stroma (fig. 4). The increased accumulation of HA on tumor cells surfaces (fig. 5) and associated stroma during early and mid-cellular differentiation of tumors (fig. 1) of different origins irrespective of tumor types was observed. Indeed, in well-differentiated tumors the enriched accumulation of hyaluronan was observed and is well in accordance with previous investigations. However some of the poorly differentiated tumors such as liver, gallbladder, prostate, endocervix, ovary, testes and osteosarcoma showed negligible presence stromal HA. The results of the present investigation are condensed in to Table 1, which shows differential HA expression between well-differentiated and poorly differentiated tumor with respect to tumor cells and tumor associated stroma.

**Table 1:** Evaluation of Hyaluronan staining in various human tumor tissues with different grades:

Tumor types	Grade	Tumor cells	Stroma
Astrocytoma	WD	XXXX	XXX
	PD	XX	XX
Ganglioglioma	WD	XXXX	XX
	PD	X	XXX
Medulloblastoma	WD	XXX	XXX
	PD	Neg.	XX
Thyroid (papillary)	WD	XXXX	XXX
	PD	Neg.	XX
Mucoepidermoid	WD	XXX	XXX
	PD	X	XX
Breast (infiltrating duct)	WD	XXXX	XXXX
	PD	X	XX
Breast (medullary)	WD	XXXX	XXXX
Lung	WD	XXX	XXX
	PD	X	XXX
Stomach	WD	XXX	XXX
	PD	X	XX
Descending colon	WD	XXX	XXX
	PD	X	XXX
Caecum	WD	XXX	XXX
	PD	Neg.	XXX
Liver	WD	XXX	XXX
	PD	Neg.	X
Gall bladder	WD	XX	XX
	PD	Neg.	Neg.
Pancreas	WD	XXX	XXX
	PD	Neg.	XX
Renal cell carcinoma	WD	XX	XXXX
	PD	X	XXX
Nephroblastoma	WD	XXX	XXX
	PD	Neg.	XX
Urinary bladder	WD	XXX	XXX
	PD	Neg.	XX
Prostate	WD	XX	XX
	PD	Neg.	XX
Endocervix	WD	XXX	XX
	PD	Neg.	X
Ovary	PD	Neg.	X
Testis	PD	Neg.	X
Osteosarcoma	WD	XXX	XXX

XXXX- very strong, XXX- strong, XX- moderate, X- weak WD-Well differentiated, PD- Poorly differentiated

The observations from our previous and present investigations as well as the observations from different laboratories around the world enticed us to speculate the existence of intricate multiple pathways towards the regulation of HA synthesis and degradation. Such pathways might involve temporal over-expression of *Has *[42]; Hyaluronidase enzyme [37,40]; and HA-binding proteins [4,9] during tumor development and progression. Evidence is mounting that suggests the extracellular matrices degrading enzymes are involved in controlling multiple steps in tumor invasion, metastasis and adhesion [33,34]. The observation that the differential clinical significance of HA and HYAL1 (Hyaluronidase 1), the correlation of Hyaluronidase enzyme in prostate tumor, its application for detecting intermediate and in high-grade bladder cancer [37] indicated the possible down regulation of HA in poorly differentiated tumors. Additionally, it was also shown that the accumulation of HA fragments is accompanied with tumor metastasis and neovascularization [18,40]. The above observations partially answered our questions about the loss of HA on the tumor cells during the progression of tumor from well differentiated through poorly differentiated stages.

There are a number of ways the high levels of HA might promote tumorigenesis. First, the increased detachment and motility of malignant cells during early and mid- stages of the tumor are due to the increased accumulation of HA, which in turn caused the deformation of restrictive architecture of the ECM components and provides a hydrated environment to facilitate migration of tumor cells to distant sites [4,28-30]. Second, increase in the interaction of HA with intracellular and surface receptors (CDC37, IHABP, RHAMM & CD44) activates the HA mediated signaling for the active invasive nature of the malignant tumor cells during early stage of tumor progression. Third, increased tumor invasion and migration may be due to increased synthesis of stromal HA through the interactions of stromal fibroblasts with tumor cells. It is possible that in addition to the above factors, there are key events such as temporal expression of metalloproteinase [31,32], the interaction between HA-HABP in early malignancy and subsequent loss of this interaction during late malignancy and the increase in stromal synthesis of HA may regulate the tumor cell behavior during the progression of tumors. The growing evidence of the presence of intracellular hyaluronan and its interaction with intracellular hyaladherins such as CDC37, IHABP4 [5,8] and further the subsequent loss of HA interaction with its receptor during the late malignancy led us to compare the distribution of H11B2C2 (one of the HA receptor detected from IVd4 hybridoma) receptor and hyaluronan expression in multiple cancer tissues in later invasive stages.

### Loss of HA in poorly differentiated tumors could be due to loss of HA, HA-receptor interaction and the over expression of H11B2C2 antigen

**Figure 6:**
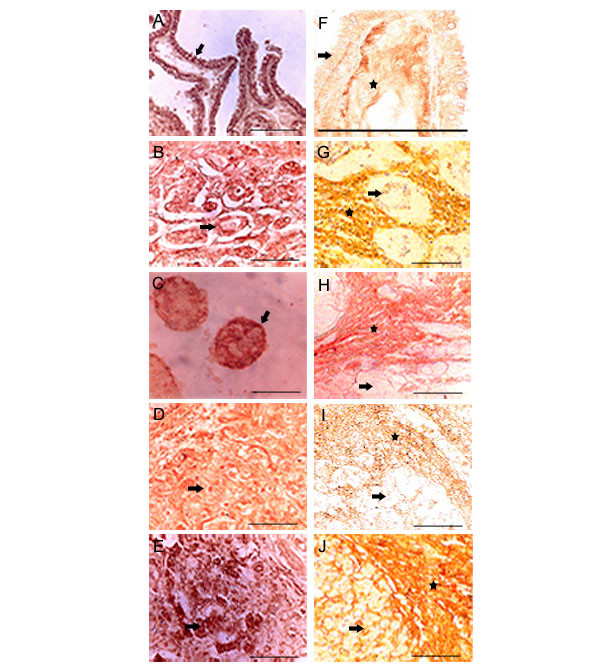
**Comparison of HABP and HA expression in poorly differentiated tumors: **HABP expression in poorly differentiated tumors **(A, B, C, D, E)**, **A. **Thyroid, **B. **Stomach, **C. **Descending Colon, **D. **Urinary bladder, **E. **Pancreas shows high HABP expression both on tumor cell surface and nucleus. HA expression in poorly differentiated tumors **(F, G, H, I, J)**, **F. **Thyroid, **G. **Stomach, **H. **Descending Colon, **I. **Urinary bladder, **J. **Pancreas shows very low HA expression in tumor cells where as the tumor stroma shows high expression of HA. Arrow shows the tumor cells and the asterisk shows stroma. Scale bars, 50 μm.

In accordance with our previous observations of the linear over expression of H11B2C2 antigen recognized by IVd4 hybridoma [5] during tumor progression [9], the present investigation showed a decline in HA expression by the tumor cells while maintaining its presence in the tumor associated stroma. The discovery of H11B2C2 protein is one more addition to the previously observed HA-binding proteins such as CDC37 and IHABP4 recognized by the same IVd4 hybridoma. The significant physiological role of these proteins in vertebrates is well explained [5,8]. In the Figure 6 we have presented six poorly differentiated tissues in an effort to demonstrate the complete loss of HA expression and continued over-expression of H11B2C2 by the tumor cells during the progression of tumor through the well differentiated to poorly differentiated stage. The left column shows strong HA expression in the stroma with reduced HA expression by the tumor cells (Fig 6-F,G,H,I and 6J). Whereas, the right column shows the over expression of H11B2C2 antigen on the tumor cells (Fig 6-A,B,C,D and 6E).

The loss of hyaluronan by the tumor cells could be due to multiple, intricate and interdependent events that takes place during the tumor progression. The increased over -expression of HA-receptor during the tumor progression might act as an interactive sink through endocytosis of highly-expressed hyaluronan by the tumor cells and its subsequent degradation by lysosomal hyaluronidase could be contributing to the loss of hyaluronan in later stages of invasive tumor cells. In support of this concept, a number of receptors including liver endothelial cell receptor [43] and lymph vessel receptor LYVE-1 homologue of CD44 have been shown to be the most efficient agents for internalization and degradation of Hyaluronic acid of any origin [44]. It has been shown, the over-expression of the receptors such as CDC37, CD44 and RHAMM, in addition to the over-expression of *Has *during early and mid differentiation of tumors [4,9,42].

Wike-Hooley [39] in 1984 observed the increased synthesis of acidic hyaluronidase, which degrades hyaluronan in the matrix and its possible correlation with tumor-aggressive potential in well differentiated tumors. It is probable that plasma derived acidic hyaluronidase were active in well differentiated tumors while the secreted hyaluronidase at near neutral activity as described in prostate [37], brain, colon carcinomas and malignant melanomas [40] was observed in highly advanced malignant tumors and even during necrosis, with the loss of hyaluronan expression. However, the continuous over-expression of HA-receptor, H11B2C2 antigen from well differentiated through poorly differentiated stage signifies its important role in clinical diagnosis of progressive tumors. Further work is in progress to understand the relation ship between synthesis of hyaluronidase, metalloproteinases with the depletion of hyaluronan and increased expression of H11B2C2 antigen in highly aggressive tumors.

## Conclusion

The present study enlighten the importance of spatial and temporal HA expression during tumor progression. It also explains the positive association of stromal HA expression with invasive nature of tumors irrespective of their origins. The present study explains the differential expression behavior of hyaluronan in well differentiated tumors in contrast to the poorly differentiated one. The loss of hyaluronan in poorly differentiated tumors could be due to internalization of hyaluronan by the HA receptors in conjunction with the increased synthesis of endogenous Hyaluronidase enzyme. Thus the interaction between HA-Hyaluronan receptor plays a significant role in tumor progression.

## Authors' contributions

RKB, HNA, MS, SBK conducted histopathological experiment; SS and SB is helped in grading human tumors; BT provided HA oligomers; SS SB, RKB, TKN and MKK helped in the evaluation of stained tissue section. SB, RKB, BT helped in the interpretation of results.
